# PReS-FINAL-2133: Drug survival and switching of biological agents in systemic juvenile idiopathic arthritis

**DOI:** 10.1186/1546-0096-11-S2-P146

**Published:** 2013-12-05

**Authors:** A Woerner, F Uettwiller, I Melki, B Bader-Meunier, R Mouy, C Wouters, P Quartier

**Affiliations:** 1 Pediatric Rheumatology, University Children's Hospital, Basel, Switzerland; 2 Department of Pediatric Immunology, Hematology and Rheumatology, Hôpital Necker-Enfants Malades, Paris, France; 3 Allergology and Clinical Immunology Unit, Clocheville Hospital, Tours, France

## Introduction

Several biologic agents have become available for the treatment of systemic juvenile idiopathic arthritis (SJIA) over the last decade. Prescription strategies may depend on disease course, which is heterogeneous and other factors including the availability of biological agents, ongoing clinical trials and National marketing authorizations.

## Objectives

To assess drug survival of biological agents in SJIA patients and to describe reasons for switching or discontinuating biologic treatment.

## Methods

A retrospective observational study was conducted on SJIA patients treated in a French pediatric rheumatology reference center using the CEMARA register, a nation-wide information system for rare diseases. We included patients who started biotherapy between 2005 and 2012 with a follow-up of at least 6 months after treatment initiation. Factors for switching or discontinuation of a biological agent were assessed.

## Results

74 SJIA patients were included, with 41 female and 33 male subjects and a median age of 4.1 years at diagnosis [range 9 months to 15.1 years]. Median disease duration before starting the first biological agent was 17.3 months [range 1.7 to 107]. The cumulative follow up on biologics represented 266.5 patient-years. Concomitant treatment included non-steroidal anti-inflammatory drugs in 94%, systemic steroids in 84% and disease-modifying anti-rheumatic drugs [7 methotrexate, 1 hydroxychloroquine, 1 leflunomide] in 12% at onset of biologic treatment. First-line biological agents were anakinra (ANA) in 45 patients, canakinumab (CAN) in 13, tocilizumab (TCZ) in 3, etanercept (ETA) in 12 and adalimumab (ADA) in 1 patient. At 3 months, drug survival for ANA versus CAN versus TCZ versus ETA as first-line biological therapy was 82% versus 100% versus 100% versus 67% percent, respectively. At 12/24 months, drug survival for ANA was 55/55%, for CAN 76/69%, for TCZ 67/67% and for ETA 58/58%, respectively. With first-line treatment, clinical remission was obtained in 55/69/67/9% of ANA/CAN/TCZ/ETA treated patients, respectively.

35/15/33/63% of ANA/CAN/TCZ/ETA treated patients switched to a second biological agent. At 3/12/24 months of second-line treatment, drug survival was 80/20/20% for ANA, 67/67/33% for CAN, 100/100/40% for TCZ and 67/67/33% for ETA. One patient was switched from ETA to abatacept with a drug survival of 73.6 months. 26% and 4% of the patients experienced a switch to a third and fourth biological agent, respectively. In total, 64% of the patients achieved clinical remission with one or up to four biological agents.

Reasons for switching treatment were lack of efficacy in 37%, adverse events in 21%, loss of response to treatment in 32% and patient's/parent's choice in 10%. Biological treatment was stopped in 10.8% of the patients due to inactive disease after a median time of 35.1 months [range 9 to 69 months]. Relapse of symptoms occurred in 42% after cessation of biotherapy.

## Conclusion

Switching to a second or third biological agent is an appropriate approach for treatment of SJIA. Median drug survival was comparable for ANA, CAN and TCZ when used as a first line biologic.

## Disclosure of interest

A. Woerner Grant/Research Support from: Novartis, Consultant for: Roche, F. Uettwiller: None declared., I. Melki: None declared., B. Bader-Meunier Grant/Research Support from: Abbvie, Chugai-Roche, Novartis, Pfizer, Consultant for: Abbott/Abbvie, BMS, Chugai-Roche, Novartis, Pfizer, Servier, SOBI, R. Mouy Grant/Research Support from: Abbvie, Chugai-Roche, Novartis, Pfizer, Consultant for: Abbott/Abbvie, BMS, Chugai-Roche, Novartis, Pfizer, Servier, SOBI, C. Wouters: None declared., P. Quartier Grant/Research Support from: Abbvie, Chugai-Roche, Novartis, Pfizer, Consultant for: Abbott/Abbvie, BMS, Chugai-Roche, Novartis, Pfizer, Servier, SOBI, Speakers Bureau: Chugai-Roche, Novartis, Pfizer.

